# RETRACTION: XBP1 Inhibits Mesangial Cell Apoptosis in Response to Oxidative Stress via the PTEN/AKT Pathway in Diabetic Nephropathy

**DOI:** 10.1002/2211-5463.13947

**Published:** 2024-12-06

## Abstract

**RETRACTION**: Y. Wang, Z. He, Q. Yang, and G. Zhou, “XBP1 Inhibits Mesangial Cell Apoptosis in Response to Oxidative Stress via the PTEN/AKT Pathway in Diabetic Nephropathy,” *FEBS Open Bio* 9, no. 7 (2019): 1249‐1258, https://doi.org/10.1002/2211‐5463.12655.

The above article, published online on 02 June 2019, in Wiley Online Library (wileyonlinelibrary.com), has been retracted by agreement between the journal Editor‐in‐Chief, Miguel A. De la Rosa; FEBS Press; and John Wiley and Sons Ltd. The retraction has been agreed upon following an investigation into concerns raised by a third party, which revealed inappropriate image duplications between this (Figure 1C‐F, 3A and C) and other articles that were previously published or published in the same or following year. Given the extent of the identified issues, the editors have lost confidence in the data presented and consider the conclusions of this manuscript substantially compromised.

**RETRACTION**: Y. Wang, Z. He, Q. Yang, and G. Zhou, “XBP1 Inhibits Mesangial Cell Apoptosis in Response to Oxidative Stress via the PTEN/AKT Pathway in Diabetic Nephropathy,” *FEBS Open Bio* 9, no. 7 (2019): 1249‐1258, https://doi.org/10.1002/2211‐5463.12655.

The above article, published online on 02 June 2019, in Wiley Online Library (wileyonlinelibrary.com), has been retracted by agreement between the journal Editor‐in‐Chief, Miguel A. De la Rosa; FEBS Press; and John Wiley and Sons Ltd. The retraction has been agreed upon following an investigation into concerns raised by a third party, which revealed inappropriate image duplications between this (Figure 1C‐F, 3A and C) and other articles that were previously published or published in the same or following year. Given the extent of the identified issues, the editors have lost confidence in the data presented and consider the conclusions of this manuscript substantially compromised.

